# Occurrence of Intestinal Parasites of Public Health Significance in Fresh Horticultural Products Sold in Maputo Markets and Supermarkets, Mozambique

**DOI:** 10.3390/microorganisms9091806

**Published:** 2021-08-25

**Authors:** Cátia Salamandane, Maria Luísa Lobo, Sónia Afonso, Regina Miambo, Olga Matos

**Affiliations:** 1 Group of Opportunistic Protozoa/HIV and Other Protozoa, Global Health and Tropical Medicine, Medical Parasitology Unit, Instituto de Higiene e Medicina Tropical, Universidade Nova de Lisboa, 1349-008 Lisboa, Portugal; catialsilvamac@gmail.com (C.S.); luisalc@ihmt.unl.pt (M.L.L.); 2 Nova School of Business and Economics, Universidade Nova de Lisboa, 2775-405 Carcavelos, Portugal; 3 Faculdade de Ciências de Saúde, Universidade Lúrio, Nampula 4250, Mozambique; 4 Department of Para-Clinics, Faculty of Veterinary Medicine, Eduardo Mondlane University, Maputo 3453, Mozambique; safonso9@hotmail.com (S.A.); regil.miambo@gmail.com (R.M.); 5 Environmental Health Institute, Faculdade de Medicina da Universidade de Lisboa, 1649-028 Lisboa, Portugal

**Keywords:** fresh horticultural products, Maputo markets, foodborne diseases, intestinal parasites, *Giardia duodenalis*, *Cryptosporidium* spp., *Enterocytozoon bieneusi*, public health

## Abstract

(1) Background: *Giardia duodenalis* and *Cryptosporidium* are important neglected parasites associated with diarrhea, such as the emerging *Enterocytozoon bieneusi*. All three are foodborne parasites raising concerns in public health. This study intended to understand the intestinal parasite occurrence with emphasis on *G. duodenalis*, *Cryptosporidium*, and *E. bieneusi* in fresh fruits/vegetables sold in the main municipal markets of Maputo city, Mozambique. (2) Methods: A total of 321 fresh horticultural products were purchased in the rainy and dry seasons (five markets/two supermarkets/one agricultural zone). Light microscopy (LM) and PCR analysis were performed. (3) Results: By LM and/or PCR, 29.3% of the samples presented at least one parasite (rainy season: 22.9%; dry season: 35.1%). The most contaminated horticultural products: collected in dry season, from Zimpeto and Fajardo markets, lettuce and pointed white cabbage. Overall, 3.7% of *G. duodenalis*, 1.3% of *E. bieneusi*, and other intestinal parasites (pathogenic and non-pathogenic) were identified. (4) Conclusions: Important pathogenic intestinal parasites were identified in fruits/vegetables commercially purchased in Maputo City. This fact must be taken into consideration when planning the management of these horticultural markets, in order to reduce the risk of contamination of fresh produce by intestinal parasites, and to prevent foodborne diseases.

## 1. Introduction

Intestinal parasites are the cause for most of the morbidity and mortality worldwide, associated with diarrhea, dysentery, intestinal obstruction, and consequently can promote a mental development retardation in children [1,2]. These parasites can be transmitted through contaminated water, soil, and food, such as fresh fruits and vegetables (when eaten raw) with pathogens infective forms (eggs, cysts, oocysts, and spores). Shellfish, fish, and meat can also transmit intestinal parasites mainly when not cooked properly. *Cryptosporidium* and *Giardia* are the most dangerous parasites causing diarrheal diseases, being responsible for cryptosporidiosis and giardiasis, respectively [3,4]. Infection with intestinal parasites is directly linked to poor hygiene and sanitation environments, and its dissemination is done through fecal-oral transmission. In fact, in developing and low-income countries, the lack of sanitary conditions and inadequate water supply and their links with the environment facilitate contamination by intestinal parasites. The clinical presentation of infections caused by intestinal parasites can vary from asymptomatic (in most cases) to severe watery diarrhea, fever, weight loss, gastrointestinal complaints, and even possible extra-intestinal manifestations [2,5,6]. In recent studies of parasites prevalence, in fresh vegetables the most reported species were: *Cryptosporidium* spp., *Giardia duodenalis*, *Cyclospora cayetanensis*, *Entamoeba* spp., *Toxoplasma gondii, Balantidium coli, Blastocystis* sp., *Cystoisospora belli*, *Enterocytozon bieneusi*, *Ascaris lumbricoides*, *Trichuris trichiura*, *Strongyloides stercoralis*, and hookworms [1,7,8]. In industrialized countries, the presence of parasites in water systems can be responsible for outbreaks [6,7]. Other factors that may contribute for parasitic infections are decreased immunity, malnutrition, age, gender, and climate [2,9,10].

The consumption of vegetables and fruits is strongly recommended by the World Health Organization for its benefits for a balanced diet and, more recently, it is a lifestyle. The intake of fruits and vegetables protect people from chronic diseases providing easily the ingestion of nutrients and vitamins that are essential for a healthy life [5,11,12]. Nevertheless, the ingestion of raw vegetables and fruits that are not properly washed can contribute significantly for foodborne diseases. Most of the fresh vegetables sold in the markets are produced in community gardens, so they are exposed to various entry points of contaminants, from production, through distribution, transport, storage, until they reach the consumer. Not only poor hygiene practices, from the producer to the sale, but also the enrichment of agricultural land with animal manure and the irrigation process with wastewater are factors that can significantly contribute to food contamination, especially horticultural products [11,13]. In addition, infective stages of intestinal parasites are present in soil and environment, and they easily pass to vegetables during the cultivation process.

In Africa, there are plenty of open markets where vegetables and fruits are sold. In these markets fresh produce is exposed to the surrounding environment, namely domestic animals that represent an additional factor for food contamination [11,13,14]. On this continent, studies on the prevalence of intestinal parasites associated with vegetables and fruits have been performed mostly in Egypt, Nigeria, Ethiopia, and Ghana. In Mozambique, a country located in Southern Africa, there are few studies about the prevalence of parasites associated with children, pets, and livestock [8,15,16,17,18,19,20]. Thus, the present study aimed to understand the occurrence of intestinal parasites in fresh horticultural products sold in the most populous municipal markets and in the two largest supermarkets in the country’s capital, Maputo, and in an agricultural zone that supplies the city, located in the peri-urban area of Maputo.

## 2. Materials and Methods

### 2.1. Study Area

This work took place in Maputo City, the capital of Mozambique in three Municipal Districts, namely Kamubukwana, Kamfpumo, and Nlhamankulo. Three types of markets were considered according to the City Council typology: a wholesale market, Zimpeto; four neighborhood markets, Central da Baixa, Fajardo, Xipamanine, and Benfica; two supermarkets, LMC and LSP, and a location within the production area (called Zona Verde/Green zone). Excluding the supermarkets (LMC and LSP) and the downtown market (Central da Baixa), the other markets are typically open.

### 2.2. Data Collection

The harvest was carried out in the dry (February to March) and rainy (August to October) seasons, 2019. Eight types of horticultural products were harvested, in triplicate, in each market. The fruits and vegetables selected were the fresh ones, which can be eaten raw and mostly used in salads. The chosen products were: coriander (*Coriandrum sativum*), parsley (*Petroselinum crispum*), Portuguese cabbage (*Brassica oleracea costata*), pointed white cabbage (*Brassica oleracea capitata*), carrot (*Daucus carota*), tomato (*Solanum lycopersicum*), green pepper (*Capsicum annuum*), and lettuce (*Lactuca sativa*). These horticultural products were purchased randomly in the morning.

### 2.3. Sample Analysis

We conducted horticultural product extract concentration and microscopy analysis. After purchase, once in the laboratory, the samples were weighed 150–250 g for large fruits/vegetables (lettuce, pointed white cabbage, and Portuguese cabbage), and 80–100 g for small ones (coriander, parsley, carrot, tomato, and green pepper). They were washed in 200 mL and 50 mL for large and small fruits/vegetables, respectively, in a plastic bag with distillate water and NaCl (0.85%). Then, they were shaken vigorously for 15 min and brined overnight. The next morning, only the solution was collected in 50 mL Falcon tubes and centrifuged at 4500 rpm for 20 min. Finally, 5–8 mL of concentrated vegetable extracts were saved and divided: 2 mL for DNA extraction, 2–4 mL for direct microscopy analysis, and the remainder for staining smears. Light microscopy was used to identify parasites in fresh smears. An aliquot of the concentrated sample was applied on a slide; a drop of Lugol’s iodine was added and then observed under a microscope with 40X amplification. An air-dried smear of the concentrated extract of each sample under study was stained by modified Ziehl–Neelsen (MZN) for identification of *Cryptosporidium* oocysts [21].

ELISA analysis. To investigate the presence of *Entamoeba histolytica*, an ELISA test based on second generation monoclonal antibody E. HISTOLYTICA II ™, Techlab (Blacksburg, VA, USA) was used for the rapid detection of *E. histolytica* adhesin in the concentrated extracts. This complementary test was applied to the positive samples (frozen concentrated extracts) obtained by light microscopy.

Molecular analysis. For DNA genomic extraction, a QIAamp Fast DNA Stool Mini Kit (50) was used, according to the manufacture’s guidelines. *G. duodenalis*, *Cryptosporidium*, and the microsporidian *E. bieneusi* DNA detection was performed by nested PCR assays, using specific primers targeting the *β*-giardin gene [22], the 18S subunit [2,23], and the entire internal transcribed spacer (ITS) region of the rRNA gene [24], respectively.

### 2.4. Social Information

Most vendors were women (80%) and the rest were men (20%). In the production field, children were involved mainly to help with irrigation and small jobs, partly because they were part of the family and because they were cheap labor. There were few animals close to the production field like chickens and dogs moving around. Farmers use chicken manure for the enrichment of their agricultural fields, without informing the horticultural products consumers about these potential risk procedures to public health.

### 2.5. Data Analysis

Samples that showed positive results with at least one of the methods (light microscopy and/or PCR-based techniques) used were considered positive for this study.

For the present study, IBM SPSS (IBM Corp, New York, NY, USA) Statistics 26 package was used to analyze the obtained data. The *t*-test for independent variables was used to compare markets with supermarkets, considering the occurrence of parasites and seasonality. All the tests were performed considering 95% (*p* = 0.05) confidence level to explain the risks to public health.

## 3. Results

Taking into account positive results by light microscopy (LM) and/or PCR based methods, 29.3% (94/321) of the samples studied presented at least one parasite, 22.9% (35/153) and 35.1% (59/168) in the rainy and dry seasons, respectively. The rate of parasitic contamination of horticultural products from each type of market was also analyzed. According to the kind of market monitored the parasite contamination rates of 31.5% (70/222), 24.7% (20/81), and 22.2% (4/18) were determined for markets, supermarkets, and agricultural zone, respectively.

According to the method used, 25.2% (81/321) of analyzed samples presented intestinal parasites, by LM. The results showed that in the rainy and dry seasons the samples collected had rates of parasites contamination of 20.3% (31/153) and 29.8% (50/168), respectively. Nested-PCR targeting *Cryptosporidium*, *Giardia* and *E. bieneusi* species identified 4.1% (13/321) positive samples, corresponding to 2.6% (4/153) in the rainy season and 5.4% (9/168) in the dry season. No *Cryptosporidium* spp. were detected by LM or PCR analysis. Table 1 summarizes the list of the horticultural products included in this study that were positive for pathogens monitored by LM and/or PCR, according to Maputo markets, during the rainy and dry seasons.

The statistical analysis revealed significant differences on the parasite’s occurrence associated with the seasonal variation, and to the type of market studied. By LM and/or PCR methods, a higher percentage of horticultural products contaminated with parasites was observed in the dry season (35.1%), both in markets and monitored supermarkets, compared to the rainy season (22.9%) (*p* = 0.000). Furthermore, a higher occurrence of parasites was observed in horticultural products from markets (31.5%) when compared to those collected in supermarkets (24.7%) (*p* = 0.000) (Annex I).

**Figure 1:**
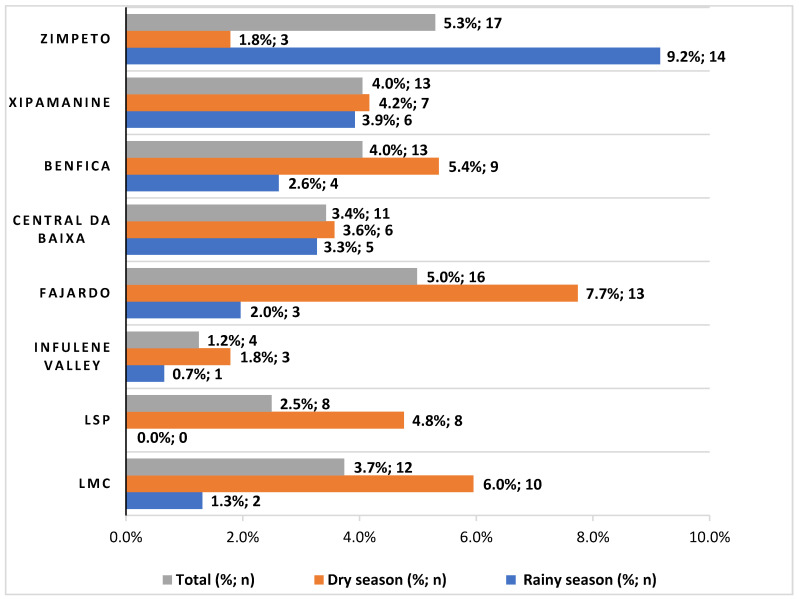
Frequency of intestinal parasites in horticultural products (*n* = 321) in markets, supermarkets and the agricultural zone of Maputo, in the rainy (*n* = 153) and dry (*n* = 158) seasons, using LM and/or PCR techniques.

As can be observed in Figure 1, during the rainy season the Zimpeto market was the one with the highest contamination (9.2%). On the other hand, Infulene Valley had the lowest contamination rate (0.7%), and, in the LSP supermarket, no contaminated horticultural products were detected (0.0%). During the dry season, the Fajardo market, the LMC supermarket and the Benfica market were the most contaminated ones with 7.7%, 6.0%, and 5.4% of contamination rate, respectively, and the least contaminated were the Zimpeto market and Infulene Valley, both with 1.8% contamination rate. Overall, the highest percentage of contaminated products was observed in the Zimpeto and Fajardo markets, with 5.3%, and 5.0%, and surprisingly, the Infulene Valley, the farm zone, had the lowest rate of contamination of horticultural products (1.2%).

**Figure 2:**
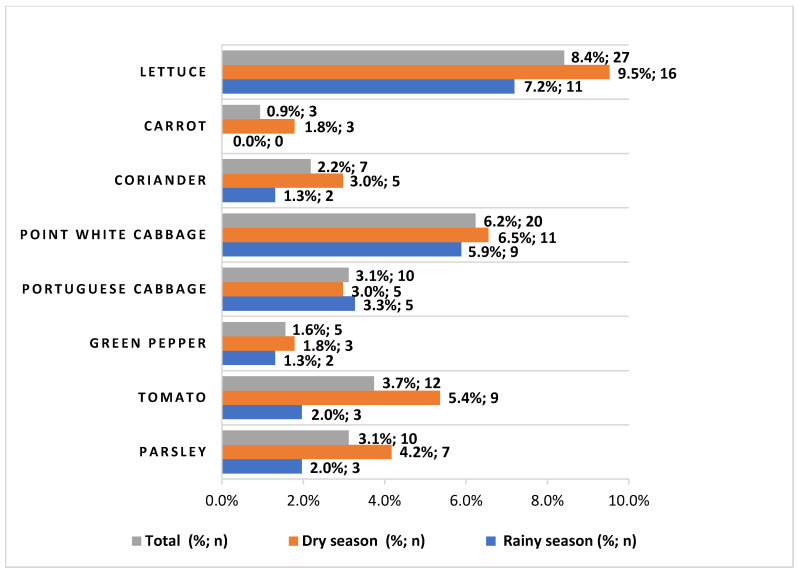
Frequency of contamination by intestinal parasites of the 321 horticultural products studied, during the rainy (*n* = 153) and dry (*n* = 168) seasons.

Overall, by the LM and/or PCR techniques, the most contaminated products analyzed were lettuce (8.4%) and pointed white cabbage (6.2%) both in the rainy and dry seasons (Figure 2). Then, followed the tomato with a high rate of contamination mainly in the dry season (5.4%) and the parsley (4.2%). Although the number of carrots harvested during the rainy season was small, the degree of contamination, in general, was the lowest (0.9%) in the studied vegetables, followed by green pepper (1.6%) and coriander (2.2%).

In the parasitized samples, one or more species concurrently were detected, by microscopy and/or PCR methods. The results of the genera/species of the pathogens identified in the 321 horticultural products by LM and/or PCR, from the Maputo markets, supermarkets and agricultural zone monitored, during rainy and dry season are summarized in Table 2.

Among the total horticultural products monitored by microscopy and/or PCR methods, *Entamoeba* spp. was the most prevalent protozoan identified with 7.8% (25/321), followed by *Entamoeba coli* with 6.5% (21/321), *Balantidium coli* 4.0% (13/321), *G. duodenalis* with 3.7% (12/321), *Entamoeba histolytica*/*E. dispar* 3.4% (11/321), *Endolimax nana* 1.6% (5/321), *Chilomastix mesnili* and *Blastocystis hominis* with 1.3% (4/321) each, *Iodamoeba butschlii* with 0.6% (2/321), and *Entamoeba hartmani* with 0.3% (1/321). The microsporidian *Enterocytozoon bieneusi* was identified in 1.3% (4/321) of the samples. Helminth eggs in general and nematodes specifically were found with a similar percentage of 1.6% (5/321) and *Strongyloides stercoralis* (adult worm) was detected in 1.3% (4/321) of the samples (Table 2).

By PCR analysis only, it was observed that 3.1% (10/321) and 1.3% (4/321) of horticultural products were contaminated with *G. duodenalis* and *E. bieneusi* (3.4%), respectively. During the dry season more than twice of the markets showed contaminated products with these species compared to the rainy season. At least one of these species was identified in all markets surveyed except for LSP supermarket (Table 2). During the rainy season, it was found that four horticultural products were positive for intestinal parasites: lettuce, green pepper (both in Zimpeto), tomato (Benfica), and pointed white cabbage (Central da Baixa). This finding increases if we use the results of microscopy. In the dry season, the positive samples detected were pointed white cabbage in Zimpeto, Fajardo, and LMC; lettuce in XP and LMC; parsley in Xipamanine and LMC; tomato in BE; and coriander in the FJ market.

The presence of pathogenic species was detected in some of the horticultural products, although with a low prevalence (ranges from 1.3% to 4.0%) (Table 2): *Balantidium coli* in lettuce, pointed white cabbage (Zimpeto), and Portuguese cabbage (Central da Baixa) during the rainy season, and in pointed white cabbage (Xipamanine), tomato (Benfica, Central da Baixa, Fajardo, and LSP) and Portuguese cabbage (Central da Baixa and Fajardo) during the dry season; *Giardia duodenalis* were mostly detected during the dry season and although appeared mainly in leafy horticultural products such as lettuce, pointed white cabbage, parsley (Zimpeto, Xipamanine, Fajardo, Infulene Valley, and LMC) it was also observed in the non-leafy vegetables green pepper (Zimpeto), tomato (Benfica), and carrot (Xipamanine). Cysts of *Entamoeba histolytica*/*E. dispar* complex were detected in lettuce (Zimpeto and Infulene Valley) and tomato (Zimpeto) during the rainy season and in coriander (Xipamanine), carrot, green pepper (Benfica), pointed white cabbage, Portuguese cabbage (Central da Baixa), and lettuce (Fajardo) in the dry season. The potentially pathogenic protozoa *Blastocystis hominis* was detected in coriander and pointed white cabbage (Zimpeto) and in tomato (Fajardo and LSP) during the rainy and dry seasons, respectively. Other pathogenic occurrence was *S. stercoralis*, which was detected only in two products, lettuce (Zimpeto and Fajardo) and parsley (LSP supermarket), during the dry season. Helminth eggs were observed in pointed white cabbage (LMC supermarket) in the rainy season, and in lettuce (Benfica and LSP) and parsley (LPS supermarket) during the dry season. Nematodes were detected only during dry season mainly in lettuce (Infulene Valley), and in carrot (Xipamanine), and coriander (LSP). Finally, spores of *E. bieneusi* were detected in the pointed white cabbage (Central da Baixa) in the rainy season, and in tomato, coriander, and lettuce (Benfica, Fajardo, and LMC supermarket, respectively) during the dry season.

**Figure 3:**
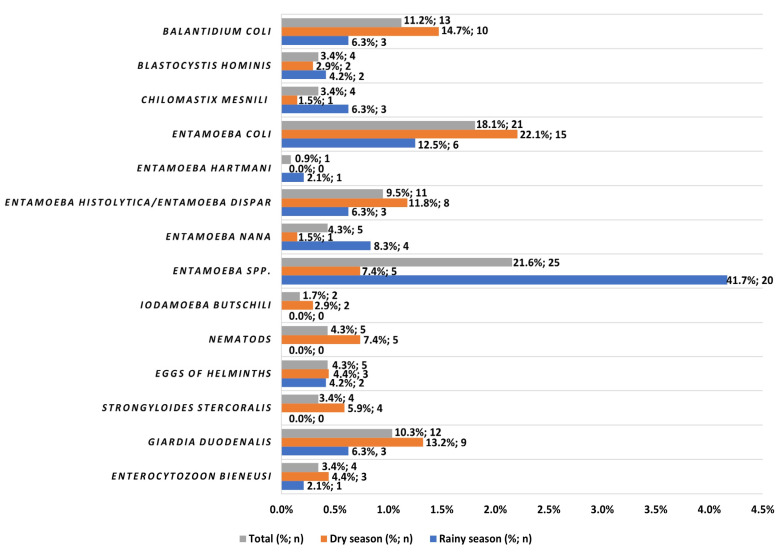
Frequency of the distinct genera/species identified among the 116 positive samples, by LM and/or PCR, and distribution according to the rainy (*n* = 48) and dry (*n* = 68) seasons.

The frequency of the distinct genera/species identified among the 116 positive cases, obtained in this study, and their distribution according to rainy and dry seasons can be observed in Figure 3.

The pathogenic species (*B, coli*, *E. histolytica/E. dispar,* nematodes, eggs of helminths, *S. stercoralis* adult worm, *G. duodenalis*, and the microsporidian *E. bieneusi*) increased their occurrence in the dry season. *Balantidium coli* was the most frequent pathogenic species (14.7%), followed by *G. duodenalis* with 13.2%, *E. histolytica*/*E. dispar* with 11.8%, and the potentially pathogenic *B. hominis* was the less frequent (2.9%) in the dry season (Figure 3).

ELISA analysis.

None of the hypothetical *E. histolytica* positive samples by LM was confirmed by the ENTAMOEBA HISTOLYTICA II™ ELISA quick test.

Ziehl Nielsen staining.

No *Cryptosporidium* spp. oocysts were observed in the smears of the horticultural products solution extracts by MZN stain. The slides were observed under a microscope at 40× magnification (three smears of each sample).

## 4. Discussion

In Mozambique there is only one study on intestinal parasites occurrence related to fresh horticultural products for human consumption, published in a Mozambican scientific journal [25]. Other reports about parasites are usually connected to human [18,26] and veterinary medicine [17].

Taking into account LM and/or PCR results we observed an overall frequency of contamination of the horticultural products of 29.3%, ranging between 22.9% (rainy season) and 35.1% (dry season), indicating a significant seasonal variation (*p* = 0.000), with the highest frequency of parasites occurrence in the dry season (in markets and supermarkets) and the lowest in the rainy season. Although contamination of horticultural products may occur in a variety of ways, it is mainly associated with contaminated water used for irrigation [27]. Our findings can be explained because during the rainy season there is no need to fetch water for irrigation, normally the Infulene Valley is flooded and there is more frequently running water for those markets that have piped water; therefore, the probability of producers and vendors washing their products well is high. Another hypothesis is assuming that parasite forms on the surface of vegetables are washed away by rain. Our data are in agreement with other authors. A study performed in Alexandria, Egypt found significantly higher rates of contaminated vegetables during spring and summer (49.3%) compared to autumn and winter (9.3%) (*p* = 0.001) [28]. Another survey conducted in Brenha, Egypt reported significant seasonal variation (*p* = 0.05) with the highest level of sample contamination detected in summer (49%) compared to winter (10.8%) [29]. Reasons indicated for this fact were: the use of untreated water/wastewater for irrigation during the warm seasons, due to the lack of rain, which is similar to what occurs in the Infulene Valley, in our study [13]; the availability in the environment of some parasites, which are more conducive to warmer climates [28,30].

Considering the results of LM and/or PCR, the most contaminated markets were Zimpeto and Fajardo with 5.3%, and 5.0%, respectively, of the products analyzed contaminated. Zimpeto presented a higher number of positive cases (9.2%) during the rainy season and Fajardo (7.7%) in the dry season. Zimpeto, which is the only wholesale market in Maputo, is also an open-air market. In this market, fresh products producers come together to sell their products, whether they are vegetables or animals. Products come from the Infulene Valley (producer zone in Maputo) [18] and the water availability and sanitary facilities conditions are not satisfactory to guarantee the overall hygiene. Fajardo market is partially covered, and it has almost the same conditions of toilet facilities as Zimpeto. Despite the better availability of water on a daily basis, the presence of all kinds of domestic small animals and the condition of the floor, which is affected by rain, as it is flooded, makes the market inadequate for displaying fresh products, increasing the probability of the occurrence of parasites. Comparing with similar studies of the same type of markets in India and at two sites in Ghana [14,31,32], the reported results describe similar and distinct high contamination rates of vegetables (8% to 12% and 18% and 67.8%, respectively).

In our study, in Maputo markets leafy vegetables were the horticultural products with the highest values of contamination. Overall, by light microscopy and/or nested-PCR techniques lettuce (8.4%) and pointed white cabbage (6.2%) were the products most contaminated with intestinal parasites, in both seasons (Figure 3). Some authors have reported even higher rates of contamination than those found in this study using light microscopy in Brazil (89%), Ghana (70.8%), Egypt (45%), Sudan (36.5%), and Norway (26%) [14,28,30,33,34]. In China and Brazil, in other studies the authors detected lettuce as the most contaminated vegetable with a contamination rate similar to ours, 7% and 8.3–13.3%, respectively, by PCR analysis [34]. Generally, high contamination rates of lettuce and other types of leafy vegetables (cabbage, parsley and coriander) are mentioned in other studies due to the uneven external surface of their leaves, which facilitates the easy entry and adherence of parasites [35]. In the present study, the lowest total contamination rate was observed in carrots (0.9%), green peppers (1.6%) and coriander (2.2%) (Figure 3). Similar results were reported in the United Arab Emirates, where the authors did not find contaminated carrots and peppers [36], and recorded contamination only for parsley (12.8%) by light microscopy. However, these results are different from those reported by Duedu et al. in Ghana [14], who found high contamination rates in carrots (28%) and green peppers (33%), also with the light microscopy technique. As for tomatoes, in our study, the total contamination rate detected was 3.7%, with 2.0% and 5.4% in the rainy and dry seasons, respectively (Figure 3), occurring in the markets of Fajardo, Zimpeto and Benfica and LMC supermarket. In tomato samples the pathogenic organisms *B. coli*, *G. duodenalis* and *E. bieneusi* were found and cases of polyparasitism were detected, which is not a surprise, since this situation arises in the most contaminated markets. Our results are in agreement with data reported by Bakri et al. for tomatoes (5.6%) in the United Arab Emirates [36]. In Sudan, the tomato contamination rate was slightly higher than ours, ranging between 9.5% and 13.3% in two different markets [3]. In turn, the Portuguese cabbage presents a total contamination rate of 3.1%, with similar percentages in both seasons. The occurrence of the pathogenic *B. coli* was observed in this vegetable in Zimpeto, Xipamanine, Fajardo and Central da Baixa markets. In addition, cysts of the *E. histolytica*/*E. dispar* complex and of commensal parasites, as *E. coli* were observed in samples of Portuguese cabbage.

Overall, the rate of occurrence of parasites in the researched horticultural products showed that some of the most frequent species are included in the *Entamoeba* group: *Entamoeba* spp. (7.8%; mainly in the rainy season), *Entamoeba coli* (6.5%), *E. histolytica/E. dispar* complex (3.4%) and *E. hartmani* (0.3%) cysts (see Table 2). Parallel to our findings, a study in Egypt detected low prevalence of *E. histolytica* in markets (3.7%) and in fields (6.4%) [27] using PCR [3,37]. Additionally, Duedu et al. [14] in Ghana found *E. histolytica* (7.0%) and *E. coli* (4.0%) in horticultural products, by microscopy. Occurrence rates of *Entamoeba* spp. higher than those we found in horticultural products in Maputo were found in markets in Sudan (42.9%), Egypt (36%) and Brazil (30.6%) [3,29,38]. Lower infection rates were found in supermarkets in Brazil (13.9%) and Sudan (14.3% of *E. histolytica*) [3,38]. All these results were obtained with the support of light microscopy. Although our results, obtained by light microscopy, suggest *E. histolytica/E. dispar* in some horticultural products, the *E. histolytica* II™ ELISA rapid test performed on those samples did not confirm the presence of this important species. Indeed, some morphological characteristics of the few cysts observed were consistent with *E. histolytica* forms, although it was not possible to validate this observation with the complementary rapid test used. Nevertheless, the absence of positive cases by ELISA may be associated with the low sensitivity of the test, which may not be sufficient to detect very low parasite loads such as those found in extracts from the samples in this study. Moreover, according to ELISA manufacturers’ protocol, the optimal results are obtained with specimens collected and analyzed in less than 24 h. If specimens are not assayed within this period of time, false negatives may occur due to adhesin degradation. In addition to the concentrated extracts having been frozen, the samples were tested several days after the indicated period, which may have affected our results. Some studies, reported the best performance of light microscopy against immunoassays to *E. histolytica* detection [39,40,41]. However, other studies have reported the difficulty of identifying *E. histolytica/E. dispar* using microscopy methods and recommend the use of the PCR technique [27,42], considering that it is very important to perform the differential diagnosis of this pathogen among the *Entamoeba* group. Molecular-based methods seem to be the best approach to overcome this problem [5,43].

*Balantidium coli*, causing balantidiosis, is a pathogen that hosts mostly in pigs but can also infect humans through contaminated water [44]. Studies on the occurrence of *B. coli* in vegetables are scarce, although, in most cases where *B. coli* was detected, it was considered the most prevalent contaminating parasite, as in the studies carried out in Cameroon (32.4%) and Ghana (13.6%) [32,33]. In this study, *B. coli* was the most prevalent pathogen (4.0%) detected in monitored horticultural products and had the third highest occurrence rate (11.2%) among all positive samples (see Figure 3). However, the occurrence rates found in our study are below the rates reported above. In the present study, lettuce, pointed white cabbage, Portuguese cabbage, and tomato were found contaminated with *B. coli,* especially these last two products, more frequently during the dry season. The occurrence of this pathogen was commonly associated with *Entamoeba* spp. or *E. coli.* Additionally, in Cameroon it was reported that all types of vegetables analyzed were contaminated with *B. coli* and *Entamoeba* spp. [33].

The following most frequent protozoan species identified among horticultural products in both seasons was *G. duodenalis* (3.7%), with a higher occurrence during dry season (Table 2). *Giardia duodenalis* was mostly identified in lettuce, pointed white cabbage, and parsley, but also in green pepper, tomato, and carrot, and some cases of polyparasitism (co-infection with *B. coli*, nematodes and mainly with parasites from *Entamoeba* group) were noticed on these products. Polyparasitism was reported in another study in lettuce and parsley [38]. Similar low rates of contamination with *G. duodenalis* were observed in Brazil, the United Arab Emirates and Ghana with 2.8%, 3.0%, and 6.0%, respectively in lettuce and cabbage mostly by microscopy [38,45,46]. Differently from our findings, there are studies that report high contamination rates (22.9%), like in Sudan [3], in products such as lettuce, green pepper, carrots, and tomato, also by microscopy.

*Endolimax nana* recorded a low overall contamination rate (1.6%), as observed for *C. mesnili* and *B*. *hominis* (1.3% each). *Chilomastix mesnili* is considered a non-pathogenic parasite, but it is rarely mentioned on this type of studies [33,43]. *Blastocysts hominis* has been more recently referred as a potentially pathogenic species commonly detected in immunocompromised individuals and associated with a variety of human gastrointestinal disorders [47]. The appearance of this parasite associated with fresh food was for first reported in Italy in ready-to-eat mixed salads (0.5%) and recently in Syria in lettuce from different markets (30.8%) and in Brazil in lettuce (15%) and coriander (19%) from markets and supermarkets [5,48,49,50].

*Enterocytozoon bieneusi* is the most prevalent microsporidian species in humans, mostly associated with infection of the intestinal tract of the patient [24]. In this study, a low frequency (1.3%) of this species was detected in PCR- monitored horticultural products, mainly during dry season. There are few reported data on the occurrence of microsporidia in fresh fruits/vegetables. A study in China [34] detected a total of 3.5% of *E. bieneusi* in some vegetables, lettuce, coriander, cucumber and chives, by molecular analysis only, but not in Chinese cabbage and leafy mustard.

Organisms belonging to Nematoda Phylum, but not clearly specified, and eggs from other helminths were detected with low-rate occurrence (1.6%), as observed for *S. stercoralis* (1.3%). Helminths are commonly reported in most of the studies monitoring intestinal parasites in vegetables worldwide. *Strongyloides* sp. eggs (5.9%) and Hookworm/*Strongyloides*/free-living nematode larvae were reported in Philippines (0.9%), in the United Arab Emirates (12.1%), in Nigeria (*S. stercoralis* [0.6%] and hookworm [1.2%]) and also in Saudi Arabia (9.2%) [30,32,36,45,51,52]. The occurrence of helminths in these studies are mostly associated with leafy and big vegetables, lettuce and cabbage. The same was registered in the present study, helminths were detected mostly in lettuce in Infulene Valley and in Fajardo market, pointed white cabbage in LMC supermarket, coriander in LSP and surprisingly in carrots from Xipamanine and parsley in LMC and LSP supermarkets, during the dry season (Table 2). Some studies [5,35] mentioned the easy way how parasites can enter these types of vegetables and adhere to their external surfaces, as we mentioned before. The higher occurrence of these parasites during the dry season, in addition to the common reasons suggested for the protozoa detected in this study, may also be related to the excretion of parasites eggs into the environment by humans or animals, with greater intensity in warm seasons compared to the cold seasons [28,53,54]. The occurrence of helminths associated with the soil and all environmental sanitary conditions, cattle manure for field enrichment, defecation in the field or even the consumption of contaminated water and its use for irrigation is also mentioned [11,14,35,55,56]. The resistance of helminth eggs to environmental factors and their occurrence mainly in small aromatic vegetables such as parsley or mint leaves has also been cited [57,58]. We also noticed *G. duodenalis* and *S. stercoralis* co-infections in some of the contaminated leafy vegetables such as lettuce and pointed white cabbage.

Despite the use of the nested-PCR technique, it was not possible to identify the pathogenic protozoan *Cryptosporidium* spp. between the samples of fruits and vegetables analyzed. This method was considered the best for the detection of *Cryptosporidium*, but it may still have limitations in biological or plant samples with very low parasite load and/or presence of factors inhibiting DNA amplification [6,59,60,61,62]. Studies carried out in Africa on vegetables and/or fruits reported similar results, and for those that detected *Cryptosporidium* spp. and *G. duodenalis* rarely exceeded 10% [32,53,63,64], and the most used method to identify these species was the microscopic analysis of vegetable solution extracts. In fact, microscopy methods have been used all over the world, mainly in developing countries, due to their efficiency in identifying parasites and continue to be important for developing countries, where molecular techniques are still very expensive and need specialized conditions that are not available in many laboratories [35,58,63,65,66,67,68].

The characteristics of the markets surveyed and already described, namely the absence of proper hygiene and sanitary conditions for products/venders/buyers could be pointed out as propitious to the occurrence of intestinal parasites (protozoans, helminths, or microsporidian) on horticultural products. However, the source of contamination could have other origins. The transport of products can represent an important risk factor for contamination of fruits/vegetables, mainly cross contamination [30]. Another important place where product contamination can occur is the production area. The Infulene Valley is the farm zone enrolled in this study that provides fresh fruits and vegetables to the markets and supermarkets of Maputo city. It is in this area that producers use manure from different origins, mainly from chicken or livestock farmers, to enrich the land, without any treatment or information given to buyers about these products in use. Another important fact associated with the contamination of horticultural products, is the type of water used for irrigation, which sometimes comes from the sewage disposal. In addition, the absence of toilet or any type of hygiene facilities or even more the inadequate water for personal hygiene [69], in this area could contribute to the occurrence of the parasites in the fruits and vegetables produced [13,19,66,70,71]. Although we observed the lowest contamination rates in the products tested in the production zone, some pathogenic species (*G. duodenalis* and nematodes) were identified.

When comparing the occurrence of parasites by the type of markets studied, a greater abundance of contaminated fruits/vegetables was observed in markets than in supermarkets, as expected. However, the frequency of contaminated products, including the pathogenic parasites, in supermarkets (24.7%), was not expected, considering all the existing strict rules and the conditions of water availability, transport, handling and display of products for their safety. Similar studies carried out in Ghana found ten times highest contamination rates in three open markets (39%, 30% and 25%) and with a large spectrum of parasites when compared to the three supermarkets studied (2%, 3% and 2%) [14], which differs from the findings of the present study. This leads us to think that the possible contamination of horticultural products in the markets and supermarkets in our study may have a similar origin, which is in agreement with other authors who report that post-harvest contamination can occur during transport and handling of the products [30]. The handling of products during preparation for display in the supermarket or even by the consumer can be one of the factors for the high level of contamination that we found [30].

In the present study, important pathogenic and non-pathogenic species of parasites were detected by LM and/or PCR analysis (Table 2). Indeed, the occurrence of several intestinal pathogens in the two main markets with the highest rates of contaminated samples is certainly associated with their specific structural and environmental characteristics, which contribute to the contamination of food products. However, in the other places of different typology surveyed, including the agricultural zone, despite the low rates of products’ contamination observed, several pathogenic species were also detected. For example, in Central da Baixa, which is a covered, well-structured market with all the guaranteed resources, availability of water, market stalls and toilets, as well as in the two supermarkets studied, similar pathogenic intestinal parasites were identified in the horticultural products sampled.

This study confirms the presence of important pathogenic intestinal parasites in the horticultural products, collected from the different types of wholesale suppliers, in Maputo city, that can be a source of human and other animal infection. This study confirms the presence of important pathogenic intestinal parasites in vegetables, collected from different types of wholesale suppliers, in the city of Maputo, which can be a source of human and other animal infection. In turn, the occurrence of non-pathogenic intestinal parasites in these products, not being, in itself, a public health problem, may be an indicator of the potential contamination of horticultural products by intestinal pathogenic parasites since both share the same fecal-oral transmission pathways. Another major concern is the fact that many people tend to consume these horticultural products raw, in salads, and other typical street food available in markets and/or nearby [72]. Thus, this study suggests a high-risk of consumption of raw horticultural products contaminated with pathogenic intestinal parasites and the need to implement better safety practices in the production, transport and handling of these products, between producers and consumers, in order to reduce their contamination and the possibility of foodborne outbreaks.

## 5. Conclusions

The present study represents a step towards understanding the occurrence of intestinal parasites in fresh horticultural products and the importance this has for public health in the metropolitan area of Maputo, Mozambique. It is indeed the first study of intestinal parasites related to fresh vegetables and fruits commercialized in Maputo markets and supermarkets using molecular biology techniques in addition to microscopy techniques. This study emphasizes the importance of good hygiene and agricultural practices at all stages of the food supply chain. Likewise, it shows that horticultural products sold in markets and supermarkets have the same probability of contamination. Although *Cryptosporidium* spp. was not detected, the occurrence of *Giardia duodenalis* (3.7%) and *Enterocytozoon bieneusi* (1.3%) added to the fact of the existence of polyparasitized horticultural products can represent an important concern for public health in this region. Furthermore, the occurrence of species of pathogenic parasites of the *Entamoeba* group and of *Balantidium coli* and even of helminths, responsible for intestinal disorders and that can acquire extra intestinal locations, requires greater public health attention. It also alerts us to the need for well-planned handling of a wide spectrum of fresh produce to reduce contamination by intestinal parasites. As such, training for good practices in each of the phases of distributing and handling fresh food from harvest to fork is mandatory.

## Figures and Tables

**Table 1:** Occurrence of intestinal parasites in the 321 samples analyzed by LM and/or PCR, collected in five markets, two supermarkets and an agricultural zone in Maputo, during the rainy and dry seasons.

Market	Horticultural Products	Rainy Season			Dry Season		
		N	Microscopy *n* (%)	PCR *n* (%)	N	Microscopy *n* (%)	PCR *n* (%)
ZP	Le	3	3 (2)	1 (0.7)	3	1 (0.6)	0 (0)
	Ca	3	0 (0)	0 (0)	3	0 (0)	0 (0)
	Co	3	2 (1.3)	0 (0)	3	0 (0)	0 (0)
	PWC	3	2 (1.3)	0 (0)	3	0 (0)	1 (0.6)
	PC	3	2 (1.3)	0 (0)	3	0 (0)	0 (0)
	GP	3	1 (0.7)	1 (0.7)	3	0 (0)	0 (0)
	To	3	1 (0.7)	0 (0)	3	0 (0)	0 (0)
	Pa	3	1 (0.7)	0 (0)	3	1 (0.6)	0 (0)
XP	Le	3	2 (1.3)	0 (0)	3	0 (0)	1 (1.2)
	Ca	NA	ND	ND	3	1 (0.6)	0 (0)
	Co	3	0 (0)	0 (0)	3	2 (1.2)	0 (0)
	PWC	3	1 (0.7)	0 (0)	3	1 (0.6)	0 (0)
	PC	3	1 (0.7)	0 (0)	3	0 (0)	0 (0)
	GP	3	0 (0)	0 (0)	3	0 (0)	0 (0)
	To	3	0 (0)	0 (0)	3	0 (0)	0 (0)
	Pa	3	2 (1.3)	0 (0)	3	1 (0.6)	1 (0.6)
BE	Le	3	2 (1.3)	0 (0)	3	2 (1.2)	0 (0)
	Ca	NA	ND	ND	3	2 (1.2)	0 (0)
	Co	3	0 (0)	0 (0)	NA	ND	ND
	PWC	3	1 (0.7)	0 (0)	3	1 (0.6)	0 (0)
	PC	3	0 (0)	0 (0)	3	0 (0)	0 (0)
	GP	3	0 (0)	0 (0)	3	2 (1.2)	0 (0)
	To	3	0 (0)	1 (0.7)	3	1 (0.6)	1 (0.6)
	Pa	3	0 (0)	0 (0)	3	0 (0)	0 (0)
CB	Le	3	1 (0.7)	0 (0)	3	2 (1.2)	0 (0)
	Ca	NA	ND	ND	NA	ND	ND
	Co	3	0 (0)	0 (0)	3	0 (0)	0 (0)
	PWC	3	1 (0.7)	1 (0.7)	3	2 (1.2)	0 (0)
	PC	3	2 (1.3)	0 (0)	3	1 (0.6)	0 (0)
	GP	3	0 (0)	0 (0)	3	0 (0)	0 (0)
	To	3	0 (0)	0 (0)	3	1 (0.6)	0 (0)
	Pa	3	0 (0)	0 (0)	3	0 (0)	0 (0)
FJ	Le	3	1 (0.7)	0 (0)	3	3 (1.8)	0 (0)
	Ca	NA	ND	ND	3	0 (0)	0 (0)
	Co	3	0 (0)	0 (0)	3	0 (0)	1 (0.6)
	PWC	3	1 (0.7)	0 (0)	3	2 (1.2)	1 (0.6)
	PC	3	0 (0)	0 (0)	3	3 (1.8)	0 (0)
	GP	3	0 (0)	0 (0)	3	0 (0)	0 (0)
	To	3	1 (0.7)	0 (0)	3	3 (1.8)	0 (0)
	Pa	3	0 (0)	0 (0)	3	0 (0)	0 (0)
IV	Le	3	1 (0.7)	0 (0)	3	3 (1.8)	0 (0)
	Ca	NA	ND	ND	3	0 (0)	0 (0)
	Co	NA	ND	ND	NA	ND	ND
	PWC	3	0 (0)	0 (0)	NA	ND	ND
	PC	NA	ND	ND	3	0 (0)	0 (0)
	GP	NA	ND	ND	NA	ND	ND
	To	NA	ND	ND	ND	ND	ND
	Pa	NA	ND	ND	3	0 (0)	0 (0)
LSP	Le	3	0 (0)	0 (0)	3	1 (0.6)	0 (0)
	Ca	NA	ND	ND	A	ND	ND
	Co	3	0 (0)	0 (0)	3	1 (0.6)	0 (0)
	PWC	3	0 (0)	0 (0)	3	1 (0.6)	0 (0)
	PC	3	0 (0)	0 (0)	3	0 (0)	0 (0)
	GP	3	0 (0)	0 (0)	3	1 (0.6)	0 (0)
	To	3	0 (0)	0 (0)	3	3 (1.8)	0 (0)
	Pa	3	0 (0)	0 (0)	3	1 (0.6)	0 (0)
LMC	Le	NA	ND	ND	3	2 (1.2)	1 (0.6)
	Ca	NA	ND	ND	NA	ND	ND
	Co	3	0 (0)	0 (0)	3	1 (0.6)	0 (0)
	PWC	3	2 (1.3)	0 (0)	3	1 (0.6)	1 (0.6)
	PC	3	0 (0)	0 (0)	3	1 (0.6)	0 (0)
	GP	3	0 (0)	0 (0)	3	0 (0)	0 (0)
	To	3	0 (0)	0 (0)	3	0 (0)	0 (0)
	Pa	3	0 (0)	0 (0)	3	2 (1.2)	1 (0.6)
	Total	153	31 (20.3)	4 (2.6)	168	50 (29.8)	9 (5.4)

Note—ZP: Zimpeto; XP: Xipamanine; BE: Benfica; CB: Central da Baixa; FJ: Fajardo; IV: Infulene Valley; LSP: SP supermarket; LMC: MC supermarket; Le: lettuce; Ca: carrots; Co: coriander; PWC: pointed white cabbage; PC: Portuguese cabbage; GP: green pepper; To: tomato; Pa: parsley; N: Total of horticultural products analyzed; *n*: number of positive samples; NA: product not available; ND: not determined.

**Table 2:** Frequency of parasite species detected by LM and/or PCR in 321 horticultural products sampled in Maputo markets and in an agricultural zone, during the rainy and dry seasons.

	Rainy Season	N = 153	Dry Season	N = 168	Total N = 321 *n* (%)
	Market (Horticultural Product)	Total *n* (%)	Market (Horticultural Product)	Total *n* (%)	
*Balantidium coli*	ZP (Le, PWC); CB (PC)	3 (2.0)	XP (PWC); BE (To); CB (PC, To); FJ (PC/2, To/2); LSP (To/2)	10 (6.0)	13 (4.0)
*Blastocystis hominis*	ZP (Co, PWC)	2 (1.3)	FJ (To); LSP (To)	2 (1.2)	4 (1.3)
*Chilomastix mesnili*	ZP (To, Pa); IV (Le)	3 (2.0)	BE (PWC)	1 (0.6)	4 (1.3)
*Entamoeba* spp.	ZP (Le/2, Co, PWC, PC/2, GP, To); XP (Le/2, PC, Pa/2); BE (Le, PWC); CB (Le, PWC); FJ (Le, PWC, To)	20 (13.1)	ZP (Pa); XP (Pa); CB (PWC); LSP (GP); LMC (PWC)	5 (3.0)	25 (7.8)
*Entamoeba coli*	ZP (To); BE (Le); CB (PC); FJ (PWC, To); IV (Le)	6 (3.9)	XP (Co); BE (Ca, GP, To); CB (Le); FJ (Le, PC, To/2); LSP (PWC, To); LMC (Le, Co, PC, Pa)	15 (8.9)	21 (6.5)
*Entamoeba hartmani*	ZP (Le)	1 (0.7)		0 (0.0)	1 (0.3)
*Entamoeba histolytica/E. dispar*	ZP (Le, To); IV (Le)	3 (2.0)	XP (Co); BE (Ca, GP); CB (PWC, PC); FJ (Le/3)	8 (4.8)	11 (3.4)
*Endolimax nana*	ZP (PC/2, GP); XP (PWC)	4 (2.6)	LMC (Le)	1 (0.6)	5 (1.6)
*Iodamoeba butschlii*		0 (0.0)	FJ (PWC/2)	2 (1.2)	2 (0.6)
*Giardia duodenalis*	ZP (Le, GP); BE (To)	3 (2.0)	ZP (PWC); XP (Le, Ca, Pa); CB (Le); FJ (PWC); IV (Le); LMC (PWC, Pa)	9 (5.4)	12 (3.7)
Nematods		0 (0.0)	XP (Ca); IV (Le/3); LSP (Co)	5 (3.0)	5 (1.6)
Helminth eggs	LMC (PWC/2)	2 (1.3)	BE (Le); LSP (Le); LMC (Pa)	3 (1.8)	5 (1.6)
*Strongyloides stecoralis* (adult worm)		0 (0.0)	ZP (Le); FJ (Le/2); LSP (Pa)	4 (2.4)	4 (1.3)
*Enterocytozoon bieneusi*	CB (PWC)	1 (0.7)	BE (To); FJ (Co); LMC (Le)	3 (1.8)	4 (1.3)
	Total positives	48 (31.4)		68 (40.5)	116 (36.1)

Note—ZP: Zimpeto; XP: Xipamanine; BE: Benfica; CB: Central da Baixa; FJ: Fajardo; IV: Infulene Valley; LSP: SP supermarket; LMC: MC supermarket; Le: lettuce; Ca: carrots; Co: coriander; PWC: pointed white cabbage; PC: Portuguese cabbage; GP: green pepper; To: tomato; Pa: parsley; *n*: number of positive samples.

## Data Availability

Not applicable.
